# Exercise Motivation and Quality of Life in Cancer Survivors: The Impact of Exercise Intervention

**DOI:** 10.3390/cancers18071119

**Published:** 2026-03-30

**Authors:** Kun-Chou Hsieh, Shyh-An Yeh, Cheng-I Hsieh, Hung-Ju Li, Yun Chen, Luo-Han Lin, Meng-Chuan Huang, Chia-Chen Chang, Yu-Ling Chen, Yu-Chieh Su

**Affiliations:** 1 Division of Thoracic Surgery, Department of Surgery, E-Da Hospital, I-Shou University, Kaohsiung 82445, Taiwan; ed101650@edah.org.tw; 2 Department of Radiation Oncology, E-Da Hospital, I-Shou University, Kaohsiung 82445, Taiwan; ed101362@edah.org.tw; 3 Department of Medical Imaging and Radiological Sciences, College of Medical Science and Technology, I-Shou University, Kaohsiung 82445, Taiwan; 4 Department of Hematology and Oncology, Taipei Medical University Hospital, Taipei 10031, Taiwan; 951007@tmuh.org.tw; 5 Division of Hematology-Oncology, Department of Internal Medicine, E-Da Hospital, I-Shou University, Kaohsiung 82445, Taiwan; 6 Service Innovation Department, Hope Foundation for Cancer Care, Taipei 10058, Taiwan; amelia@ecancer.org.tw (Y.C.); rubylin@ecancer.org.tw (L.-H.L.); 7 Department of Public Health and Environmental Medicine, Kaohsiung Medical University, Kaohsiung 80708, Taiwan; mechhu@kmu.edu.tw; 8 Department of Physical Education, National Taiwan University of Sport, Taichung 40404, Taiwan; 9 Graduate Institute of Medicine, College of Medicine, I-Shou University, Kaohsiung 82445, Taiwan; 10 School of Medicine, College of Medicine, I-Shou University, Kaohsiung 82445, Taiwan

**Keywords:** cancer survivors, exercise intervention, quality of life, exercise motivation, relative autonomy index (RAI)

## Abstract

Background/Objectives: Cancer survivors often experience physical decline, fatigue, and reduced quality of life (QoL) following cancer treatment. Exercise is an important strategy in survivorship care; however, the role of exercise motivation in sustaining exercise behavior and improving QoL remains unclear. This study evaluated the effects of a structured exercise intervention on QoL, fatigue, and exercise motivation among cancer survivors. Methods: This single-arm longitudinal pre–post-intervention study recruited adult cancer survivors (median age: 55 years) with heterogeneous cancer types from E-Da Hospital, Taiwan, between October 2023 and July 2024. Participants completed a 3-month supervised exercise program consisting of weekly 60 min sessions that included aerobic, resistance, balance, and flexibility training. Assessments were conducted at baseline, immediately after the intervention, and at a 3-month follow-up. Outcome measures included physical fitness tests, fatigue scales, and the European Organisation for Research and Treatment of Cancer Quality of Life Questionnaire (EORTC QLQ-C30), and the Relative Autonomy Index for exercise motivation. Results: Thirty-nine participants completed the study. Improvements in several QoL domains were observed at the 3-month follow-up, particularly in physical and emotional functioning. Pain and fatigue symptoms decreased following the intervention, although some fatigue indicators increased again during follow-up. Female participants showed greater improvements in certain physical fitness measures. Intrinsic exercise motivation increased after the intervention, and greater motivation gains were associated with larger improvements in QoL. Conclusions: Participation in a structured exercise program may be associated with improvements in QoL, fatigue symptoms, and exercise motivation among cancer survivors. However, given the single-arm design and small sample size, these findings should be interpreted cautiously. Larger randomized controlled studies are needed to confirm the long-term benefits of exercise interventions in cancer survivorship care.

## Simple Summary

Cancer survivors often experience fatigue, reduced physical function, and poor quality of life after treatment. Although exercise is known to provide physical and psychological benefits, maintaining regular physical activity can be challenging for many survivors, partly due to low motivation. This study examined whether participation in a structured exercise program was associated with changes in quality of life and exercise motivation among cancer survivors. Participants completed a three-month supervised exercise program and were followed for an additional three months. The findings showed improvements in quality of life and reductions in pain and fatigue after the intervention. Importantly, participants who showed greater improvements in exercise motivation tended to experience larger improvements in quality of life. However, some fatigue symptoms reappeared during follow-up, suggesting that continued exercise support may be necessary. These findings highlight the potential importance of exercise programs that not only improve physical fitness but also strengthen exercise motivation, supporting exercise as an important component of cancer survivorship care.

## 1. Introduction

With the rapid increase in global cancer incidence and continuous advances in cancer detection and treatment, the number of cancer survivors has risen substantially worldwide. The number of newly diagnosed cancer cases worldwide has increased substantially over recent decades, and approximately one in five individuals is expected to develop cancer during their lifetime [1,2]. In Taiwan, cancer incidence has also risen significantly, and cancer has remained the leading cause of death for decades, imposing substantial psychological and economic burdens on patients, families, and healthcare systems [3,4,5]. Although survival outcomes have improved, many cancer survivors experience long-term physical and psychological sequelae that negatively affect quality of life (QoL). Common post-treatment complications include cancer-related fatigue (CRF), sarcopenia, reduced physical function, depression, and anxiety, all of which can impair daily functioning and overall well-being [6,7,8,9]. Previous studies have shown that cancer survivors often report significantly poorer physical and mental health compared with the general population, highlighting the need for effective survivorship care strategies that address long-term health outcomes beyond cancer treatment [6,10].

Among lifestyle interventions, regular physical exercise has been consistently shown to improve physical function, reduce fatigue, and enhance psychological well-being in cancer survivors [11,12,13]. International consensus guidelines recommend that cancer survivors engage in regular moderate-intensity aerobic exercise combined with resistance training to improve cardiovascular fitness and overall QoL [13]. In addition, regular physical activity has been associated with reduced treatment-related symptoms and improved long-term health outcomes, including a reduced risk of recurrence in certain cancer types [13]. Despite these well-established benefits, adherence to regular exercise remains relatively low among cancer survivors. Barriers such as fatigue, psychological distress, and reduced motivation frequently limit sustained participation in physical activity [14,15,16]. Therefore, identifying factors that influence long-term adherence to exercise interventions has become an important topic in cancer survivorship research.

Exercise motivation has emerged as a key determinant of sustained engagement in physical activity. Self-Determination Theory (SDT) provides a widely used theoretical framework for understanding motivation and behavioral regulation in health behaviors [17]. According to SDT, motivation exists along a continuum ranging from externally controlled motivation to intrinsic motivation, with more autonomous forms of motivation being associated with stronger behavioral persistence, greater psychological well-being, and more sustainable health behaviors [17]. In the context of exercise interventions, autonomy-supportive environments may promote internalization of motivation and encourage long-term adherence to physical activity. Previous studies have shown that such environments can enhance intrinsic motivation and improve both exercise adherence and QoL in cancer survivors [18]. However, cancer-related symptoms, treatment burden, and psychological stress may reduce motivation and make it challenging for survivors to maintain regular exercise participation.

Although the benefits of exercise for improving physical and psychological outcomes in cancer survivors have been widely documented, relatively little attention has been paid to the motivational mechanisms underlying these benefits. In particular, limited evidence exists regarding how exercise interventions influence motivational regulation and how changes in exercise motivation may be associated with improvements in QoL among cancer survivors. A better understanding of these motivational processes may help inform the development of survivorship programs that promote sustainable exercise behavior and long-term well-being.

The Relative Autonomy Index (RAI) is commonly used to assess the degree to which exercise behavior is internally regulated within the SDT framework [17,18]. Higher RAI scores indicate greater internalization of exercise motivation and stronger self-determined behavior. Therefore, evaluating changes in RAI may provide insight into how exercise interventions influence motivational processes. This present study aimed to evaluate the effects of a structured exercise intervention on QoL, fatigue, and exercise motivation in cancer survivors. In addition, we examined whether changes in exercise motivation were associated with improvements in QoL. By examining both clinical outcomes and motivational changes, this study seeks to provide evidence that may help inform exercise-based survivorship care strategies aimed at promoting sustainable health behaviors among cancer survivors [19,20].

## 2. Materials and Methods

### 2.1. Study Design and Participant Recruitment

This study was designed as a single-arm longitudinal pre–post-intervention study and was approved by the Institutional Review Board (IRB) of the E-Da Hospital, Taiwan (IRB number: EMRP12112N; approved on 28 September 2023). All participants provided written informed consent prior to enrollment. Participant recruitment was conducted from 13 October 2023 to 31 July 2024. Participants were recruited from the Hematology-Oncology outpatient clinic at E-Da Hospital, Taiwan, and the Obstetrics and Gynecology outpatient clinic at E-Da Dachang Hospital, Taiwan. A total of 48 participants were initially enrolled after screening based on the inclusion and exclusion criteria. During the intervention period, nine participants withdrew due to physical discomfort or scheduling conflicts. As of 31 December 2024, 39 participants completed the 3-month follow-up assessment and were included in the final analysis.

Given the exploratory nature of this pilot study, a formal sample size calculation was not performed prior to recruitment. The study aimed to explore the potential effects of an exercise intervention on motivation, fatigue, and quality of life among cancer survivors. The inclusion criteria were as follows: (1) age ≥ 20 years; (2) patients with cancer who had completed their primary cancer treatment (e.g., surgery, chemotherapy, or radiotherapy) and were in a stable clinical condition; and (3) an Eastern Cooperative Oncology Group (ECOG) performance status of 0–1. The exclusion criteria were as follows: (1) patients deemed unsuitable for exercise by their physician; (2) pregnant or breastfeeding women; (3) individuals unable to provide informed consent or safely participate in an exercise program.

### 2.2. Exercise Intervention

The exercise intervention consisted of a 12-session supervised exercise program conducted over a 3-month period, with participants attending one session per week. Each session lasted approximately 60 min and was supervised by trained instructors to ensure exercise safety. Each session included warm-up activities, aerobic exercise, resistance training, balance training, and cool-down stretching. The exercises were designed to improve muscle strength, balance ability, flexibility, coordination, and cardiorespiratory endurance in cancer survivors. The training program incorporated a variety of exercise activities, including chair-based exercises, body-weight resistance exercises, core stabilization training, agility exercises, and interval-based aerobic movements. These activities progressively targeted different muscle groups, including the upper limbs, lower limbs, and trunk muscles, while also promoting functional movement and coordination. The supervised sessions were intended to provide structured exercise guidance and encourage participants to maintain regular physical activity according to their individual health conditions and physical capacity. Attendance was recorded for each session to monitor participant adherence to the exercise program.

### 2.3. Assessment Time Points

Outcome assessments were conducted at three time points: (1) baseline (pre-test)—before the start of the intervention; (2) post-intervention assessment—immediately after completion of the 3-month intervention; (3) follow-up assessment—conducted 3 months after the intervention to evaluate the sustainability of the effects. The measurement schedule is summarized in Table S1.

### 2.4. Outcome Measures

The primary outcomes of this study were improvements in QoL and CRF. The secondary outcomes included changes in exercise motivation, the sustainability of exercise behavior (assessed using the Cancer Patient Exercise Behavior Barriers and Facilitators Survey), and physical fitness indicators, including muscle endurance, cardiopulmonary endurance, and balance performance.

### 2.5. Assessment Tools and Measurement Criteria

This study used both questionnaires and physical fitness tests for data collection, as detailed below:

(1) EORTC QLQ-C30

A validated Traditional Chinese version of the EORTC QLQ-C30 (version 3) was used in this study with permission from the EORTC Quality of Life Group. This questionnaire assesses the overall QoL of cancer patients, including physical, role, emotional, cognitive, and social functioning. Each item is rated on a 4-point Likert scale (1 = Not at all, 4 = Very much), while the global health status score is rated on a 7-point scale (1 = Very poor, 7 = Excellent). Scores were calculated according to the EORTC scoring manual. For ease of interpretation, all scores were oriented such that higher values indicate better functioning or quality of life. Specifically, symptom scale scores were reversed so that higher scores represent lower symptom burden. The EORTC QLQ-C30 has been widely validated as a reliable instrument for assessing QoL and cancer-related symptoms in cancer populations [21].

(2) Taiwanese version of the Brief Fatigue Inventory (BFI-T)

Cancer-related fatigue was assessed using the Taiwanese version of the Brief Fatigue Inventory (BFI-T). The BFI-T is a validated instrument adapted from the original BFI to measure fatigue severity and its impact on daily functioning in cancer patients. The instrument consists of nine items rated on a 0–10 Likert scale, evaluating fatigue severity and its interference with daily activities. Higher scores indicate greater fatigue severity. Fatigue severity is categorized as mild (1–4), moderate (5–6), and severe (7–10). The BFI-T has demonstrated good reliability and validity in Taiwanese cancer populations [22].

(3) Cancer Patient Exercise Behavior Barriers and Facilitators Survey

The Cancer Patient Exercise Behavior Barriers and Facilitators Survey (internal questionnaire, not publicly available) was used to evaluate exercise habits, barriers, facilitators, and exercise motivation.

(3.1) Exercise Motivation Assessment

Exercise motivation was assessed using 24 items representing six types of motivation based on SDT: amotivation, external regulation, introjected regulation, identified regulation, integrated regulation, and intrinsic motivation. Items were rated on a 7-point Likert scale (1 = Strongly disagree, 7 = Strongly agree). The Relative Autonomy Index (RAI) was calculated to assess the degree of self-determined motivation. Higher RAI scores indicate greater internalization of exercise motivation. RAI was calculated as follows:

RAI = Intrinsic Motivation × 2 + Integrated Regulation × 1 + Identified Regulation × 1 + Introjected Regulation × 0 + External Regulation × (−1) + Amotivation × (−2)

(3.2) Exercise Behavior Assessment

Exercise behavior items included exercise intensity (high, moderate, low), frequency (sessions per week), and duration (minutes per session).

(4) Physical Fitness Test

Physical fitness was assessed using several functional tests (internal assessment protocols, not publicly available). These included (1) Body composition: weight, body mass index (BMI), and body fat percentage; (2) Balance ability: static balance test, single-leg stance test (eyes open/closed), and Y-balance reach test; (3) Proprioception and coordination: blindfolded stepping test; (4) Cardiorespiratory endurance: three-minute step test; and (5) Muscular endurance: leg muscle endurance test. These assessments were used to evaluate participants’ physical function and exercise capacity.

### 2.6. Statistical Analysis

Descriptive statistics were used to summarize participant characteristics and outcome measures. Continuous variables were presented as mean ± standard deviation (SD). Within-participant changes across the three time points (baseline, post-intervention, and 3-month follow-up) were analyzed using repeated-measures ANOVA (IBM SPSS Statistics version 24.0, IBM Corp., Armonk, NY, USA). Assumptions of normality and sphericity were evaluated prior to conducting repeated-measures ANOVA. When overall differences were observed, post hoc pairwise comparisons with Bonferroni correction were performed. To further explore the relationship between exercise motivation and intervention outcomes, participants were stratified into two groups based on the median change in RAI (ΔRAI). The median value (ΔRAI = 18) was used as the cut-off to ensure a relatively balanced sample size between groups: improved motivation group (ΔRAI > 18) and non-improved motivation group (ΔRAI ≤ 18). A *p*-value < 0.05 was considered statistically significant. All statistical analyses were conducted using IBM SPSS version 24.0 (IBM Corp., Armonk, NY, USA). Given the single-arm longitudinal design with repeated measurements, outcome assessors were not blinded to the assessment time points.

## 3. Results

### 3.1. Participant Characteristics

A total of 48 participants were enrolled in this study, of whom 39 completed the 3-month follow-up assessment. Their baseline characteristics are summarized in Table 1. The majority of participants were female (69.2%), with a median age of 55 years (range = 30–85 years). The 46–60 years age group was the most common (41.0%). The mean BMI was 24.3 ± 5.3, with 46.2% classified as having a normal weight (18.5 ≤ BMI < 24) and 43.6% as overweight or obese (BMI ≥ 24). Stage III cancer was the most prevalent (33.3%), followed by Stage II (28.2%). The mean time from initial cancer diagnosis to the initiation of the exercise intervention was 46.9 months (SD = 51.2), with a median of 31.9 months (Q1 = 15.5, Q3 = 54.2). Most participants were married (76.9%) and lived with a spouse or family members (89.7%). Employment status was evenly distributed between those with full-time jobs (41.0%) and those who were unemployed (41.0%). In terms of education level, 33.3% had a university degree, and 12.8% held a graduate or doctoral degree. Participants attended a mean of 9.4 ± 2.3 sessions out of the 12 supervised exercise sessions, corresponding to an overall attendance rate of 78%. Using a cutoff of 9 sessions (≥75% attendance), 27 participants (69%) were classified as high-adherence, whereas 12 participants (31%) were classified as low-adherence, with the lowest attendance being 4 sessions. Adherence was monitored throughout the program to ensure participant engagement and safety.

### 3.2. Primary Outcomes

#### 3.2.1. Quality of Life Assessment (EORTC QLQ-C30)

Detailed statistical results are presented in Table 2. No significant changes were observed in the global health status score across the three time points (baseline, post-intervention, and 3-month follow-up).

For the functional scales, physical functioning showed a significant improvement at the 3-month follow-up (97.6 ± 4.2) compared with both baseline (90.6 ± 10.5, *p* < 0.001) and post-test (90.1 ± 10.5, *p* < 0.001). Emotional functioning also significantly improved at the 3-month follow-up (91.2 ± 13.2) compared with baseline (84.0 ± 21.6, *p* = 0.024). No significant differences were observed for role functioning, cognitive functioning, or social functioning.

For the symptom scales, pain scores significantly improved from baseline (81.8 ± 24.2) to post-test (90.6 ± 13.7, *p* = 0.015) and remained improved at the 3-month follow-up (91.5 ± 20.9, *p* = 0.006, compared with baseline). Fatigue also showed improvement at the 3-month follow-up (86.9 ± 15.9) compared with baseline (79.5 ± 19.8, *p* = 0.012). Dyspnea significantly improved at follow-up (96.6 ± 10.2) compared with post-test (88.0 ± 16.2, *p* = 0.006). Other symptom domains, including insomnia, appetite loss, constipation, diarrhea, and financial difficulties, did not show significant changes over time.

#### 3.2.2. Quality of Life by Cancer Stage

Among early-stage cancer survivors (Stage I & II, *n* = 17), the total QoL score at the 3-month follow-up (92.9 ± 5.0) was significantly higher than at baseline (89.7 ± 6.6, *p* = 0.024) and post-test (87.5 ± 8.6, *p* = 0.011). However, among late-stage cancer survivors (Stage III & IV, *n* = 18), no significant changes in QoL scores were observed between different time points (*p* > 0.05). There were no significant differences in QoL improvements between early-stage and late-stage groups (*p* > 0.05) (Table S2).

#### 3.2.3. Fatigue Severity Assessment (BFI-T)

The fatigue assessment results are shown in Table 3. The current level of fatigue was significantly lower at the post-test (1.85 ± 1.95) compared with baseline (2.56 ± 2.26, *p* = 0.044). However, the fatigue level at the 3-month follow-up (2.51 ± 2.40) was similar to the baseline value (*p* = 0.919). Similarly, the worst fatigue level during the previous 24 h was significantly reduced at post-test (2.41 ± 2.49) compared with baseline (3.51 ± 2.53, *p* = 0.001), but increased again at follow-up (4.15 ± 3.05), showing a significant difference compared with post-test (*p* < 0.001). Regarding fatigue interference, general activity showed significantly higher scores at the 3-month follow-up (2.58 ± 2.40) compared with post-test (1.51 ± 1.73, *p* = 0.009) and baseline (1.49 ± 1.89, *p* = 0.014). No significant differences were observed for other domains, including mood, walking ability, normal work, social relationships, and enjoyment of life.

### 3.3. Secondary Outcomes

#### 3.3.1. Changes in Exercise Motivation (Cancer Patient Exercise Behavior Barriers and Facilitators Survey)

**Figure 1:**
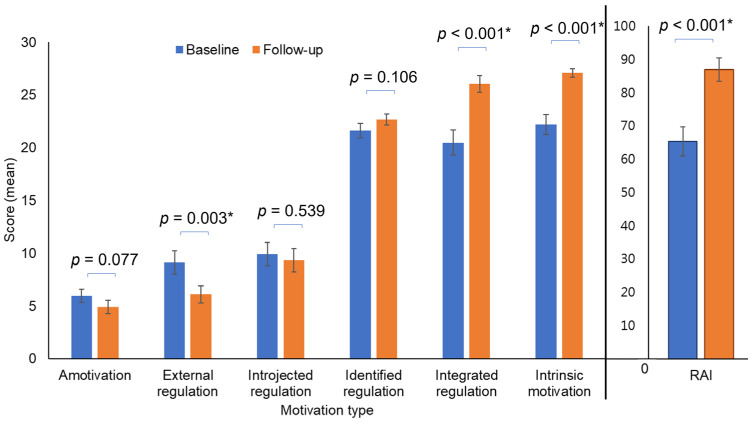
Changes in exercise motivation. * *p* < 0.05 indicates significant differences.

As shown in Figure 1, RAI increased significantly from 65.5 to 87.1 after the intervention (*p* < 0.001), indicating a shift from externally controlled to more self-determined motivation. Participants exhibited significant reductions in external regulation (9.1 ⟶ 6.1, *p* = 0.003) and increases in intrinsic motivation (22.2 ⟶ 27.1, *p* < 0.001), suggesting greater enjoyment and satisfaction from exercise. These results indicate that participants showed a shift toward more self-determined forms of exercise motivation after the intervention.

#### 3.3.2. Association Between Changes in Exercise Motivation and Quality of Life

The difference in RAI before and after the exercise intervention was defined as ΔRAI. Participants were classified into two groups based on the median ΔRAI value (18): the non-improved motivation group (ΔRAI ≤ 18) and the improved motivation group (ΔRAI > 18). The study then examined whether QoL improved in the improved motivation group compared to the non-improved motivation group. Table 4 shows that the quality of life in the improved motivation group was significantly higher at follow-up compared to the pre-test score (87.4 ± 10.1 vs. 82.7 ± 10.5, *p* = 0.022). In contrast, the non-improved motivation group showed a slight increase in QoL at follow-up, but the change was not statistically significant (90.7 ± 7.0 vs. 93.1 ± 5.9, *p* = 0.150). Notably, the pre-test QoL score of the non-improved motivation group was significantly higher than that of the improved motivation group, which may be because participants in this group already had higher exercise motivation (RAI) at baseline (Table S3). This may suggest that participants with higher baseline exercise motivation were already engaging in regular physical activity, which may have limited the magnitude of further improvement. In contrast, participants in the improved motivation group exhibited a significant increase in exercise motivation after the intervention and reached a similar RAI level to the non-improved motivation group at follow-up (*p* = 0.300), along with a more noticeable improvement in QoL (*p* = 0.022). These results suggest that participants who experienced greater increases in exercise motivation tended to show greater improvements in QoL; however, this association should be interpreted cautiously due to baseline differences and the potential influence of regression to the mean.

Further analysis in Table S3 shows that the non-improved motivation group had a significantly higher baseline RAI compared to the improved motivation group (82.7 ± 20.6 vs. 49.1 ± 22.8, *p* < 0.001). This indicates that participants in the non-improved motivation group already had high exercise motivation before the intervention, suggesting they might have maintained a consistent exercise routine, resulting in smaller changes in ΔRAI. However, the improved motivation group (ΔRAI > 18) had a lower RAI at baseline but caught up with the non-improved motivation group at follow-up (90.6 ± 10.4 vs. 83.3 ± 29.1, *p* = 0.300). This suggests that participants in this group experienced a significant increase in exercise motivation after the intervention, reaching levels similar to those in the non-improved motivation group. The results indicate a possible increase in internalization of exercise motivation, leading to sustained exercise behavior and QoL improvement

#### 3.3.3. Exercise Behavior (Survey on Barriers and Facilitators of Exercise Behavior in Cancer Patients)

Figure S1 illustrates changes in participants’ exercise behavior before and after the trial. Participants who had no prior exercise habits but adopted one after the trial were classified as having a “positive change”, while those who had an exercise habit before the trial but ceased exercising afterward were classified as having a “negative change”. The results showed that 79% (31/39) of participants maintained their exercise habits before and after the trial. Only one participant (3% = 1/39) had no exercise habit at both time points, and no participants exhibited a negative change. Among the 31 participants who maintained their exercise habit, 15 participants (48%) increased their exercise volume (either by increasing exercise intensity or extending weekly exercise duration). 5 participants (16%) maintained the same exercise volume (no change in exercise intensity or weekly duration). 11 participants (36%) decreased their exercise volume (reduced exercise intensity or shortened weekly exercise duration).

#### 3.3.4. Physical Fitness (Physical Fitness Assessment)

The results of the physical fitness assessment are presented in Table S4. The overall physical fitness score showed no significant difference between the pre-test (13.12 ± 2.69) and post-test (13.34 ± 3.08) (*p* = 0.583). When stratified by gender, female participants showed a modest but statistically significant increase in the overall physical fitness score (13.31 ± 2.30 vs. 14.12 ± 2.47, *p* = 0.017), whereas no significant change was observed among male participants. No significant differences were observed when stratified by cancer stage. Although the overall improvement in physical fitness was not statistically significant, these findings provide preliminary descriptive information regarding potential functional responses to the exercise intervention.

## 4. Discussion

This study evaluated the effects of a structured exercise intervention on physical fitness, fatigue levels, QoL, and exercise motivation in cancer survivors. From September 2023 to July 2024, a total of 39 cancer survivors completed a 3-month supervised exercise program. The results showed improvements in several QoL domains, particularly physical and emotional functioning, along with reductions in pain and fatigue symptoms. However, these findings should be interpreted cautiously because the present study was conducted using a single-arm pre–post design without a control group, which limits the ability to establish causal relationships between the intervention and the observed outcomes. Despite these limitations, the findings suggest that participation in a structured exercise program may be associated with improvements in QoL among cancer survivors. These findings are consistent with previous studies suggesting that regular exercise may improve physical functioning, reduce symptom burden, and enhance overall well-being in cancer survivorship populations [23,24]. Nevertheless, the magnitude of improvement appeared to be smaller among participants with advanced-stage cancer, which may reflect greater disease burden and treatment-related complications that can limit responsiveness to exercise interventions.

The present study also observed a significant increase in intrinsic exercise motivation following the intervention. This finding is consistent with the framework of SDT, which proposes that supportive environments can facilitate the internalization of exercise behaviors and promote more autonomous forms of motivation. Previous research by Behzadnia et al. demonstrated that autonomy-supportive exercise settings can enhance psychological well-being and promote sustained exercise participation among cancer survivors [18]. In line with SDT, autonomous and intrinsic forms of motivation have been shown to be positively associated with sustained physical activity participation in cancer survivors [25]. Systematic reviews have further highlighted that more autonomous motivation predicts greater exercise adherence and long-term physical activity participation across diverse populations [26]. Similarly, qualitative research applying SDT in men with prostate cancer has shown that autonomy-supportive environments and need-supportive exercise guidance can enhance motivation and facilitate sustained engagement in physical activity [27]. Increased intrinsic motivation may therefore play an important role in supporting long-term adherence to physical activity in cancer survivorship populations. Consistent with this perspective, Courneya et al. reported a strong association between regular exercise and improved QoL in endometrial cancer survivors, likely mediated by improvements in physical function and overall health status [28].

With respect to fatigue, participants showed improvements immediately after the intervention; however, some fatigue indicators increased again at the 3-month follow-up. In addition, general activity levels were lower at follow-up compared with baseline. One possible explanation is that the structured exercise sessions ended after the intervention period, and participants may not have maintained the same level of physical activity afterward. These findings suggest that the benefits of short-term supervised exercise programs may diminish over time without continued exercise engagement. Therefore, ongoing exercise support or longer-term exercise programs may be necessary to sustain improvements in fatigue management among cancer survivors. Previous studies have also demonstrated that exercise interventions can improve HRQoL and reduce fatigue among cancer survivors [29]. However, sustained physical activity appears to be necessary to maintain long-term improvements in cancer-related fatigue and health-related quality of life [30].

Although the intervention focused on improving physical fitness, overall improvements in objective fitness measures were not statistically significant in the full cohort, with more pronounced improvements observed among female participants. This may be partially explained by the relatively small sample size and the limited number of male participants, which restricts the statistical power for subgroup analyses. In addition, baseline physical fitness levels among some participants may have approached moderate levels, potentially limiting the magnitude of measurable improvement. The relatively low frequency of supervised sessions (once per week) was a deliberate design choice to provide structured and manageable guidance for cancer survivors who may face barriers to initiating regular exercise. However, this frequency may be insufficient to induce substantial physiological adaptations. The supervised sessions were therefore intended primarily to introduce safe exercise practices, build exercise confidence, and encourage participants to gradually incorporate additional physical activity into their daily routines according to their physical capacity. Because of the relatively small sample size in this pilot study, additional correlation analyses between changes in objective fitness measures and QoL or motivation outcomes were not performed. Future studies with larger samples may further explore these relationships. In addition, the composite physical fitness score used in this study was derived from multiple functional components (e.g., strength, balance, and flexibility), which may have limited its sensitivity to detect changes in specific domains. This may also have contributed to the lack of statistically significant improvement observed in the overall fitness score.

Another important observation is that participants in the improved-motivation group had lower baseline RAI and QoL scores compared with those in the non-improved group. Therefore, part of the observed improvement may reflect regression toward the mean, which should be considered when interpreting the association between changes in exercise motivation and QoL.

Overall, these findings highlight the potential role of exercise programs in cancer survivorship care, particularly in supporting exercise motivation and improving aspects of QoL. However, further research using larger samples and randomized controlled designs is needed to confirm these findings and better understand the mechanisms linking exercise motivation and health outcomes in cancer survivors.

### Limitations

This study has several limitations. First, the absence of a control group limits the ability to determine whether the observed improvements were directly attributable to the exercise intervention rather than other factors such as natural recovery or increased attention from study participation. Second, the relatively small sample size limits statistical power and may reduce the generalizability of the findings. Third, the heterogeneity of cancer types and disease stages may have introduced variability in treatment history and baseline health status. Fourth, potential confounding factors, including age, sex, BMI, and cancer stage, were not adjusted for in the analyses due to the study design and limited sample size. In addition, given the evaluation of multiple outcomes, there is a potential risk of type I error, and the findings should be interpreted with caution. As this was an exploratory pilot study, the results are intended to identify potential trends and generate hypotheses rather than to provide definitive conclusions. Future studies with larger sample sizes, longer follow-up periods, and randomized controlled designs are warranted to validate and extend these findings.

## 5. Conclusions

The findings of this pilot study suggest that participation in a structured exercise program may be associated with improvements in quality of life, fatigue symptoms, and exercise motivation among cancer survivors. Improvements were particularly observed in physical and emotional functioning, although some fatigue indicators increased again during follow-up, highlighting the importance of sustained physical activity for long-term fatigue management. In addition, the intervention was associated with increased intrinsic exercise motivation, supporting the role of autonomy-supportive exercise environments in promoting long-term exercise adherence. However, given the single-arm study design and relatively small sample size, these findings should be interpreted cautiously. Future randomized controlled trials with larger and more diverse populations are needed to further examine the long-term effects of exercise interventions and the role of motivation in improving survivorship outcomes.

## Figures and Tables

**Table 1:** Baseline demographic and clinical characteristics of participants.

Variables	n (%)	Variables	n (%)
Gender		Cancer stage	
Male	12 (30.8)	I	6 (15.4)
Female	27 (69.2)	II	11 (28.2)
Age (years)		III	13 (33.3)
30–45	10 (25.6)	IV	5 (12.8)
46–60	16 (41.0)	Unknown/None	4 (10.3)
61–85	13 (33.3)	Marital status	
Height (cm)	162.2 ± 8.0	Married	30 (76.9)
Weight (kg)	64.3 ± 16.3	Unmarried	9 (23.1)
BMI ^1^	24.3 ± 5.3	Living status	
Underweight (BMI < 18.5)	4 (10.3)	With spouse/family	35 (89.7)
Normal weight (18.5 ≤ BMI < 24)	18 (46.2)	Living alone	3 (7.7)
Unnormal:		Institutional care	1 (2.6)
Overweight (24 ≤ BMI < 27)	8 (20.5)	Education level	
Mild obesity (27 ≤ BMI < 30)	6 (15.4)	Primary school or below	3 (7.7)
Moderate obesity (30 ≤ BMI < 35)	1 (2.6)		
Severe obesity (BMI ≥ 35)	2 (5.1)	Junior high school	4 (10.3)
Employment status		Senior high school	9 (23.1)
Full-time	16 (41.0)	Associate degree	5 (12.8)
Part-time	3 (7.7)	Bachelor’s degree	13 (33.3)
Self-employed	4 (10.3)	Master’s/Doctoral degree	5 (12.8)
Unemployed	16 (41.0)		

^1^ BMI—Body Mass Index.

**Table 2:** Changes in quality of life measured by the EORTC QLQ-C30 at baseline, post-test, and 3-month follow-up.

Domain	Item No.	BL	Post	FU	BL vs. Post	BL vs. FU	Post vs. FU
Global health status	29–30	71.8 ± 19.1	73.9 ± 21.6	71.4 ± 18.2	0.497	0.893	0.453
Functioning Scales							
Physical	1–5	90.6 ± 10.5	90.1 ± 10.5	97.6 ± 4.2	0.776	<0.001 *	<0.001 *
Role	6–7	91.5 ± 19.1	94.0 ± 11.8	91.9 ± 19.4	0.453	0.912	0.482
Emotional	21–24	84.0 ± 21.6	89.0 ± 12.0	91.2 ± 13.2	0.102	0.024 *	0.373
Cognitive	20, 25	85.5 ± 16.3	86.8 ± 13.3	88.9 ± 13.4	0.570	0.160	0.257
Social	26–27	87.6 ± 17.0	87.6 ± 17.4	91.9 ± 14.7	1.000	0.160	0.185
Symptom Scales							
Fatigue	10, 12, 18	79.5 ± 19.8	82.9 ± 13.9	86.9 ± 15.9	0.249	0.012 *	0.080
Nausea and vomiting	14–15	98.3 ± 5.1	96.6 ± 9.5	98.3 ± 8.4	0.253	1.000	0.160
Pain	9, 19	81.8 ± 24.2	90.6 ± 13.7	91.5 ± 20.9	0.015 *	0.006 *	0.797
Dyspnea	8	90.6 ± 15.2	88.0 ± 16.2	96.6 ± 10.2	0.373	0.051	0.006 *
Insomnia	11	74.4 ± 30.1	76.1 ± 22.9	73.5 ± 31.7	0.720	0.831	0.570
Appetite loss	13	95.7 ± 11.3	93.1 ± 15.6	95.7 ± 17.4	0.262	1.000	0.446
Constipation	16	87.2 ± 21.1	87.2 ± 18.1	90.6 ± 21.6	1.000	0.253	0.253
Diarrhea	17	94.0 ± 15.0	93.2 ± 13.6	96.6 ± 10.2	0.744	0.324	0.160
Financial difficulties	28	87.2 ± 21.1	88.0 ± 17.9	89.7 ± 20.5	0.800	0.520	0.487

Abbreviations: BL—baseline; FU—follow-up. Values are presented as mean ± standard deviation (SD). Pairwise comparisons between time points were performed using repeated-measures ANOVA with Bonferroni adjustment. * *p* < 0.05 indicates statistical significance. Post hoc pairwise comparisons were performed with adjustment for multiple testing.

**Table 3:** Fatigue severity assessment (BFI-T): Statistical analysis of baseline, post-test, and 3-month follow-up.

Measure	BL	Post	FU	BL vs. Post	BL vs. FU	Post vs. FU
Fatigue Items						
Fatigue right now	2.56 ± 2.26	1.85 ± 1.95	2.51 ± 2.40	0.044 *	0.919	0.135
Usual fatigue in last 24 h	2.67 ± 2.47	1.97 ± 2.15	2.69 ± 2.42	0.069	0.954	0.142
Worst fatigue level in last 24 h	3.51 ± 2.53	2.41 ± 2.49	4.15 ± 3.05	0.001 *	0.184	<0.001 *
Interference Items						
General activity	1.49 ± 1.89	1.51 ± 1.73	2.58 ± 2.40	0.940	0.014 *	0.009 *
Mood	1.59 ± 1.92	1.38 ± 2.14	1.36 ± 2.06	0.562	0.555	0.942
Walking ability	1.41 ± 2.20	1.44 ± 2.00	1.21 ± 2.13	0.934	0.629	0.439
Normal work	2.36 ± 2.25	1.74 ± 2.11	2.38 ± 1.79	0.083	0.954	0.076
Relations with other people	1.38 ± 1.79	1.13 ± 1.82	1.05 ± 1.86	0.439	0.343	0.812
Enjoyment of life	1.10 ± 1.70	1.21 ±1.88	1.54 ± 2.30	0.753	0.267	0.346

Abbreviations: BL—baseline; FU—follow-up. Values are presented as mean ± standard deviation (SD). Overall *p*-values were calculated using repeated-measures analysis. * *p* < 0.05 indicates statistical significance. Post hoc pairwise comparisons were performed with adjustment for multiple testing.

**Table 4:** Quality of life comparison by RAI group.

Group	BL	FU	Within-Group *p*-Value
Improved motivation group	82.7 ± 10.5	87.4 ± 10.1	0.022 *
Non-improved motivation group	90.7 ± 7.0	93.1 ± 5.9	0.150
Between-Group *p*-value	0.009 *	0.039 *	

Abbreviations: BL—baseline; FU—follow-up. Values are presented as mean ± standard deviation (SD). Within-group comparisons were performed using repeated-measures analysis. Between-group comparisons at each time point were conducted using independent-samples tests. * *p* < 0.05 indicates statistical significance. Post hoc pairwise comparisons were performed with adjustment for multiple testing.

## Data Availability

The original contributions presented in this study are included in the article/supplementary material. Further inquiries can be directed to the corresponding authors.

## Supplementary Materials

The following supporting information can be downloaded at https://www.mdpi.com/article/10.3390/cancers18071119/s1: Table S1: Study timeline; Table S2: Quality of life assessment by cancer stage; Table S3: RAI comparison between non-improved and improved motivation groups; Table S4: Physical fitness assessment results; Figure S1: Changes in exercise habits.

## Abbreviations

The following abbreviations are used in this manuscript:

BFI	Brief Fatigue Inventory
BMI	Body Mass Index
CRF	Cancer-Related Fatigue
ECOG	Eastern Cooperative Oncology Group
HRQoL	Health-Related QoL
IRB	Institutional Review Board
QoL	Quality of Life
RAI	Relative Autonomy Index
SD	Standard Deviation
SDT	Self-Determination Theory
