# Supplementary material for: Exercise Motivation and Quality of Life in Cancer Survivors: The Impact of Exercise Intervention
Source: Cancers (Basel). 2026 Mar 30;18(7):1119. doi: 10.3390/cancers18071119 (PMC13072272; doi:10.3390/cancers18071119)
Supplement: Supplementary file 1 [file cancers-18-01119-s001.zip › cancers-4194776-supplementary.pdf]

Supplementary Information

Table S1. Study timeline

Assessment	Screening Phase	Pre-test Phase	Intervention Phase	Post-test Phase	3-Month Follow-up
**Informed consent explanation**	X				
**Signing of informed consent**	X				
**Physical fitness test**		X		X	
**Exercise intervention (12 sessions, 3 months)**			X		
**Questionnaire - Taiwanese version of the Brief Fatigue Inventory (BFI-T)**		X		X	X
**Questionnaire - EORTC QLQ-C30**		X		X	X
**Questionnaire – Cancer Patient Exercise Behavior Barriers and Facilitators Survey**		X			X

Table S2. Quality of life assessment by cancer stage

Cancer stage	BL	Post	FU	BL vs Post	BL vs FU	Post vs FU
Stage I, II (n=17)	89.7 ± 6.6	87.5 ± 8.6	92.9 ± 5.0	0.334	0.024*	0.011*
Stage III, IV (n=18)	86.2 ± 10.5	88.4 ± 8.4	89.6 ± 10.7	0.196	0.151	0.518
p-value (by stage)	0.243	0.749	0.247			

Abbreviations: BL, baseline; FU, follow-up. Values are presented as mean ± standard deviation (SD). Overall p-values were calculated using repeated-measures analysis. *$p < 0.05$ indicates statistical significance. Post hoc pairwise comparisons were performed with adjustment for multiple testing.

Table S3. RAI comparison between non-improved and improved motivation groups

	Non-improved motivation group	Improved motivation group	*p*-value
RAI_Baseline	82.7 ± 20.6	49.1 ± 22.8	<0.001*
RAI_Follow-up	83.3 ± 29.1	90.6 ± 10.4	0.300

Note: Values are presented as mean ± standard deviation (SD). *$p < 0.05$ indicates statistical significance.

Table S4. Physical fitness assessment results

**Group**	**Baseline**	**Post-test**	***p*-value**
Overall (n = 39)	13.12 ± 2.69	13.34 ± 3.08	0.583
Gender			
Male (n=12)	12.68 ± 3.48	11.59 ± 3.68	0.100
Female (n=27)	13.31 ± 2.30	14.12 ± 2.47	0.017*
p-value (Gender comparison)	0.508	0.016*	
Cancer Stage			
Stage I, II (n=17)	13.07 ± 2.01	13.18 ± 3.03	0.520
Stage III, IV (n=18)	13.49 ± 3.26	13.67 ± 3.31	0.767
p-value (Stage comparison)	0.643	0.651	

Note: Values are presented as mean ± standard deviation (SD). *$p < 0.05$ indicates statistical significance.

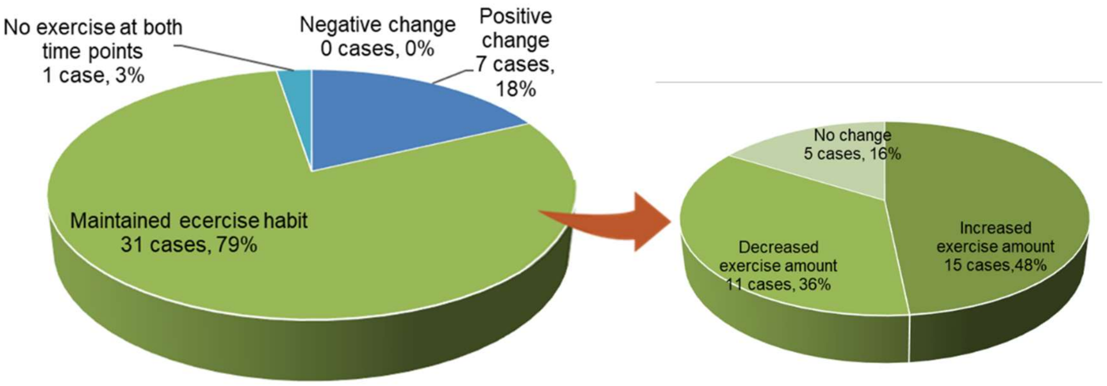

Figure S1. Changes in exercise habits.
